# Breast cancer vaccines: from immune promise to the prospect of eradication

**DOI:** 10.1097/MS9.0000000000004373

**Published:** 2025-11-14

**Authors:** Areej Imtiaz, Tayyaba Abdullah, Salman Hassan, Mahnoor Fatima

**Affiliations:** a Department of Medicine, Nishtar Medical College, Nishtar Medical University, Multan, Pakistan; b Department of Medicine, Shaheed Ziaur Rahman Medical College, Rajshahi Medical University, Bogura, Bangladesh

*Dear Editor,*

Breast cancer is the most frequently diagnosed malignancy among women worldwide, responsible for nearly 25% of all female cancer diagnoses. The global cancer statistics from GLOBOCAN 2020 data highlight its scale, with 2.3 million new cases reported and representing 11.7% of all cancer cases[1]. According to levels of human development, in very high-HDI countries, about 1 in 12 women develop breast cancer, but only 1 in 71 dies from it. In low-HDI regions, the risk of diagnosis is lower (1 in 27), yet mortality is higher, with 1 in 48 women dying of the disease. It is also found that the incidence of cancer increases with increasing HDI, and a significant positive correlation was observed between HDI and the incidence of cancer, but this was not significantly related to the death[2]. Socioeconomic status is directly related to the stage of the cancer and the patient’s survival. With the increasing incidence rate of cancer, the mortality rate from cancer does not necessarily increase. This may be due to earlier detection and treatment in developed countries. These disparities highlight the persistent global gap in breast cancer control and outcomes. In addition to its clinical significance, breast cancer is a condition with deep social and economic ramifications. It affects women often in their most productive years, it disrupts families, strains healthcare systems, and imposes long-term consequences on communities and national economies[3]. This was written in accordance with the TITAN guideline checklist 2025[4].

Although awareness and therapies have improved, the rising global burden of breast cancer emphasizes the need for prevention-focused solutions. As traditional therapies focus mainly on management after diagnosis, scientific attention has now shifted toward prevention through immune-based approaches such as cancer vaccines. Unlike prophylactic vaccines that protect against infectious diseases, breast cancer vaccines are being developed primarily as therapeutic agents. After decades of reliance on surgery, chemotherapy, and radiation to manage disease once it appears, the possibility of preventing breast cancer through vaccination marks a fundamental shift[5]. Several types of breast cancer vaccines have been investigated, including peptide-based vaccines (such as NeuVax/E75, GP2, and AE37 targeting HER2), MUC1-based vaccines, dendritic cell vaccines, and newer DNA or RNA vaccines encoding tumor antigens. Recent progress in vaccine development offers new hope, positioning immunoprevention as a potential breakthrough in reducing the future burden of this disease. If ongoing trials confirm safety and long-term efficacy, breast cancer vaccines could complement existing screening programs and be integrated into public health policies worldwide.

The rationale behind these vaccines is to harness the immune system to recognize and destroy tumor cells. Unlike traditional vaccines that target pathogens, cancer vaccines stimulate immunity against tumor-associated antigens found mainly on malignant cells[5]. One targeted antigen is α-lactalbumin, a breast-specific self-protein that is “retired” from expression in the aging female breast, is highly expressed in triple-negative breast cancer (TNBC) but absent in most normal adult tissues outside of lactation. High α-lactalbumin expression is observed in most TNBC cell lines and 72% of primary TNBC tumors, comparable to levels found in human lactating adenomas. Preclinical data support α-lactalbumin vaccination as a safe and effective preventive strategy. Human T cells responded effectively *in vitro*, and vaccinated mice were protected from breast tumors without autoimmune complications. Unlike conventional therapies or immunotherapy for advanced disease, this vaccine targets prevention. The widespread presence of α-lactalbumin in TNBC supports its potential to shift oncology from treatment to prevention[5]. Because α-lactalbumin is stably and widely expressed in TNBC, vaccines targeting it could benefit both sporadic breast cancer patients. This emerging strategy reflects the broader success of immunology in oncology and signals a future in which protection may replace treatment as the central pillar of breast cancer control^[5,6]^.

The restricted expression of α-lactalbumin makes it a safe and selective target for breast cancer vaccines, reducing autoimmunity while inducing immune attack against malignant cells. Other antigens, including HER2/neu and MUC1, have also been explored in various subtypes. Immunoprevention offers protection for high-risk women, such as those with BRCA mutations, by activating the immune system before cancer develops. A Phase I trial of an α-lactalbumin vaccine reported significant immune responses in the majority of patients with minimal side effects, supporting its feasibility and safety[7]. The goal of breast cancer vaccines is to train the immune system to target precancerous or early malignant cells that carry mutations. Early phase clinical studies, including a separate Phase I trial of 35 high-risk TNBC women, demonstrated that more than 75% developed robust immune responses, with only mild injection-site irritation[8]. Overall, these insights highlight the promise of vaccines to transform breast cancer prevention, with the potential to work alongside screening and lifestyle interventions to lessen global disparities in outcomes.

Similarly, a plasmid DNA vaccine targeting the HER2 intracellular domain demonstrated both safety and immunogenicity, by delivering DNA fragments that stimulate host cells to express HER2 antigens. This process subsequently triggers cytotoxic and helper T-cells to launch a specific immune response, with participants exhibiting potent T-cell activity and experiencing minimal adverse effects[9]. Furthermore, a study on neoantigen DNA vaccines in TNBC patients found that 14 out of 18 participants developed neoantigen-specific immune responses, and 16 remained cancer-free after 3 years, as these vaccines encode patient-specific tumor neoantigens that train the immune system to selectively recognize and eliminate malignant cells, suggesting the potential of personalized vaccine strategies for preventing cancer recurrence[10]. Beyond conventional targets, these vaccines stimulate both cytotoxic (CD8+) T-cells, which directly destroy tumor cells, and helper (CD4+) T-cells, which direct and sustain the immune reaction. Together, they create a powerful defense system that not only eliminates cancer cells but also forms enduring immune memory, enabling the body to rapidly recognize and fight tumor recurrence. This long-lasting immune response allows the body to detect malignant cells if they appear in the future. Moreover, research is exploring the combination of vaccines with checkpoint inhibitors to overcome tumor-induced immune suppression, which may enhance effectiveness in aggressive or poorly immunogenic TNBC subtypes^[8,9]^. These complementary strategies highlight the evolving role of immunoprevention as part of a broader, precision-based approach to breast cancer management.

These findings underscore the contrast with traditional treatments, including surgery, chemotherapy, radiation, and hormone therapy, which are implemented once the disease has developed. Immunotherapies like checkpoint inhibitors are generally reserved for advanced-stage disease. In contrast, vaccines provide a proactive strategy, aiming to stimulate the immune system to recognize and eliminate precancerous or early malignant cells before they develop into full-blown cancer. If validated in larger, randomized trials, these vaccine strategies could transform oncology from a treatment-focused paradigm to one centered on prevention, potentially reducing the incidence and mortality of breast cancer globally.

Breast cancer vaccines in early phase trials have generally been well tolerated. Most reported adverse events are mild and transient, such as injection-site pain, fatigue, and low-grade flu-like symptoms. Serious adverse effects are rare, reflecting the targeted mechanism of action of these therapies[11]. However, the long-term safety and durability of immune responses remain uncertain, as current data are limited to small, early phase studies with brief follow-up. Most trials have enrolled highly selected populations, prompting questions about efficacy and tolerability in diverse ethnic groups and individuals with comorbidities[12]. Finally, the feasibility, scalability, and cost-effectiveness of implementing such vaccines, especially in low- and middle-income countries, have yet to be fully addressed. These limitations underline the need for broader multicenter research to assess both safety and real-world implementation[13].

Several Phase 2 and Phase 3 clinical trials are currently underway to evaluate the efficacy and immunogenicity of breast cancer vaccines in broader patient cohorts[11]. Peptide-based, DNA, and mRNA vaccines represent the main platforms being tested, with the purpose of establishing persistent and targeted anti-tumor immune responses. Despite encouraging early outcomes, several critical questions remain unanswered. It is still unclear which populations stand to benefit most, whether those with inherited mutations such as BRCA, those at high familial risk, or cancer survivors. Moreover, the strength and longevity of vaccine-induced immunity, the role of booster doses, and the comparative performance of different platforms are subjects of ongoing investigation^[14,15]^. Addressing these uncertainties will be essential to establish the preventive value of breast cancer vaccines and to guide their integration into future cancer control strategies.

Although modern therapies have extended survival in breast cancer patients, recurrence remains a critical issue. Vaccines seek to overcome this by inducing the immune system for antitumor responses. Yet, trials with different vaccine types like DNA, peptide, and dendritic cell based have produced modest results, but have achieved a limited success due to immune complexity and tumor heterogeneity. Despite these setbacks, integrating vaccines with checkpoint inhibitors, HER2-targeted antibodies, and tumor-selective vaccines points offer renewed hope for the successful integration of vaccines into breast cancer care[16]. Ensuring that all populations can access these strategies is vital to reduce disparities and maximize the health benefits of these emerging breast cancer prevention strategies. Continued scientific investigation, policy reinforcement, and awareness initiatives will be essential to implement these innovations and significantly reduce breast cancer incidence worldwide.

Breast cancer vaccines stand at the forefront of a paradigm shift in oncology, offering new hope for prevention and long-term disease control. While early phase data highlight safety and immunogenicity, critical gaps in efficacy, durability, and optimal patient selection demand rigorous investigation through large, multicenter trials. Equally important are considerations of cost-effectiveness and accessibility, with special urgency in LMICs where the burden of disease remains highest. If these scientific and implementation challenges are successfully addressed, breast cancer vaccines could progress from trial concepts to essential elements of worldwide cancer control, moving us closer to the goal of eradication.

## Data Availability

Not applicable to this study.

## Research registration unique identifying number

N/A.
